# On Flipping Edge Sets in Unique Sink Orientations

**DOI:** 10.1007/s00373-025-02929-2

**Published:** 2025-04-25

**Authors:** Michaela Borzechowski, Simon Weber

**Affiliations:** 1 Freie Universität Berlin, Berlin, Germany; 2 ETH Zürich, Zürich, Switzerland

**Keywords:** Unique sink orientation, Reconfiguration, Phase

## Abstract

A *unique sink orientation* (USO) is an orientation of the *n*-dimensional hypercube graph such that every non-empty face contains a unique sink. We consider the only known connected *flip graph* on USOs. This flip graph is based on the following theorem due to Schurr: given any *n*-dimensional USO and any one dimension $i\in [n]$, the set $E_i$ of edges connecting vertices along dimension *i* can be decomposed into equivalence classes (so-called *phases*), such that flipping the direction of any $S\subseteq E_i$ yields another USO if and only if *S* is the union of some of these phases. In this paper we provide an algorithm to compute the phases of a given USO in $O(n\cdot 3^n)$ time, significantly improving upon the previously known $O(n\cdot 4^n)$ trivial algorithm. We also show that the phase containing a given edge can be flipped using only *poly*(*n*) space additional to the space required to store the USO. We contrast this by showing that given a boolean circuit of size *poly*(*n*) succinctly encoding an *n*-dimensional USO, it is $\textsf{PSPACE}$-complete to determine whether two given edges are in the same phase. Finally, we also prove some new results on the structure of phases.

## Introduction

A *unique sink orientation* (USO) is an orientation of the *n*-dimensional hypercube graph, such that for each non-empty face of the hypercube, the subgraph induced by the face contains a unique sink, i.e., a unique vertex with no outgoing edges.

USOs were originally proposed by Stickney and Watson [20] as a way of modeling the candidate solutions of instances of the *P-matrix Linear Complementarity Problem* (P-LCP). Since then, many more problems in geometry, game theory, as well as mathematical programming have been reduced to the problem of finding the global sink of a unique sink orientation [3, 6–9, 12, 18]. An algorithm to find the sink of an *n*-dimensional USO running in time *poly*(*n*) would imply strongly polynomial-time algorithms for both Linear Programming [9] as well as for the P-LCP; the existence of which are both major open questions. Motivated by this, much research has gone into finding better algorithms for the sink-finding problem [11], however the original algorithms by Szabó and Welzl [21] with runtimes exponential in *n* are still the best known.

While the algorithmic aspects of USOs are undoubtedly those most motivated by applications, research into structural and combinatorial aspects can be crucial for algorithmic developments. In this paper we consider one of the standard viewpoints used to study families of combinatorial objects, namely that of *reconfiguration*.

In reconfiguration, one defines simple (often “local”) operations which can be applied to the combinatorial objects under study. This defines a so-called *flip graph*, whose vertices represent the combinatorial objects, where neighboring objects can be turned into each other by applying such a simple operation. Flip graphs are particularly interesting when they are connected, or even Hamiltonian. If the flip graph is connected, enumeration of all objects can be performed by systematically walking through a spanning tree of the flip graph, e.g., with a technique due to Avis and Fukuda named *reverse search* [1]. By simulating a random walk on the flip graph (often expressed as a *Markov chain*), we can also perform random sampling among the objects. If the flip graph is furthermore Hamiltonian, we can even find a so-called *Gray code*, that is, a way to enumerate the objects such that any two consecutive objects differ only by a single application of the simple operation. Flip graphs have been studied for many types of combinatorial objects, which has in many cases contributed to a better understanding of the objects themselves [13, 16].

On USOs, a natural choice of operation is to flip the orientation of a single edge. However, it is known that there exist USOs in which no single edge can be flipped without destroying the USO condition. This follows from Keller’s conjecture on cube tilings being wrong [2, 18], which was first proven by Lagarias and Shor [14]. In other words, not only is the flip graph based on single edge flips *not* connected, it even has isolated vertices. We thus need to consider operations that may flip multiple edges at once.

Ideally, the operations we base our flip graph on should be somewhat “local”. Since we have seen that flipping single edges (the most local operation possible) does not work, the next idea is to flip a set of edges together, where all of these edges are of the same dimension, i.e., they run along the same axis of the hypercube. In his PhD thesis [18], Schurr characterized exactly which such sets can be flipped without destroying the USO condition. Schurr proved that for any USO *O* and any dimension *i*, the set $E_i$ of edges of dimension *i* can be decomposed into equivalence classes, such that whenever we flip a set $S\subseteq E_i$ in *O*, the resulting orientation is a USO if and only if *S* is the union of some of these equivalence classes. Schurr named these equivalence classes within $E_i$ the *i*-*phases* of *O*.

Based on these insights, we can now very easily define a flip graph on the *n*-dimensional USOs: The operation is to pick any dimension $i\in [n]$, compute the *i*-phases, and to flip any subset of them. Schurr showed that with only 2*n* of these operations we can obtain any *n*-dimensional USO from any other. The flip graph is thus connected and has rather low diameter. Furthermore, Schurr proved that the naturally defined Markov chain based on this operation converges to the uniform distribution.

The phase flip graph due to Schurr is the only connected flip graph for USOs that has been studied in the literature. We therefore think that it is crucial to understand this flip graph better. The open questions concerning this flip graph fall into two major areas, *structure* and *algorithms*. On the structural side, we wonder: Is the flip graph Hamiltonian? How quickly does the associated Markov chain converge? On the algorithmic side we ask: How efficiently can the Markov chain be implemented? What is the complexity of computing the phases of a given USO?

In this paper, we mostly make progress on the algorithmic side. However, we also point out several surprising structural properties of phases. We believe that these may be useful in the future for showing structural properties of the phase flip graph itself.

### Results

On the algorithmic side, we begin with some positive results in Sect. 4. We provide an algorithm to compute all phases of a given *n*-dimensional USO using $O(3^n)$ vertex comparisons, improving upon the currently best known method due to Schurr which takes $O(4^n)$ comparisons. We also show that (at the cost of higher run-time) we can flip the phase containing a given edge using only *poly*(*n*) space additional to the space used to store the USO. We contrast this second result by the following hardness result proven in Sect. 5.

#### Theorem 1.1

Given a USO *O* in succinct circuit representation, and two edges $e,e'$ of *O*, the problem of deciding whether *e* and $e'$ are in the same phase is $\textsf{PSPACE}$-complete, even if *O* is guaranteed to be acyclic.

To prove the above theorem, we actually require some more insights on the structure of phases, which are introduced first in Sect. 3. Specifically, in Sect. 3.1, we show that for every phase *P*, the subgraph of the hypercube induced by the endpoints of the edges in *P* is *connected*. In Sect. 3.2, we prove that flipping a *matching* in a USO leads to another USO if and only if the matching is a union of phases. The proof of this statement is highly non-trivial and requires the use of newer results on *pseudo USOs* [4]. Schurr previously claimed to prove this statement in his PhD thesis [18, Proposition 4.9]. However, his proof of the “only if” direction contained a severe mistake that was not possible to fix.

We provide two further structural insights in Sect. 3.3 and Sect. 3.4, however we did not find any direct applications of these insights. In Sect. 3.3, we show that to compute the phases of an *n*-dimensional USO by Schurr’s method, it is not sufficient to compare only neighboring edges or even only edges of some bounded distance. We construct a family of USOs in which one needs to compare antipodal vertices with each other. In Sect. 3.4, we prove some connections between phases and *hypervertices*. Hypervertices are faces in which for every vertex the orientation of the edges leaving the face is the same, and they have been used for a certain USO construction by Schurr and Szabó [19].

### Discussion and Related Work

*Exponential Runtime.* At first glance, an $O(n\dot{3}^n)$ time algorithm for computing the phases of an *n*-dimensional USO does not sound very efficient. The reader may worry about the practicality of a Markov chain in which already taking a single step requires exponential time. However, we would like to point out that the number of *n*-dimensional USOs is $2^{\Theta (2^n\log n)}$ [15], i.e., doubly exponential. This also shows that even the most efficient encoding of USOs uses $\Theta (2^n\log n)$ bits in the worst case. We can see that therefore our runtime of $O(n\cdot 3^n)$ is polynomial in the encoding size of a single USO and logarithmic in the number of vertices of the flip graph. This falls in line with flip graphs on other combinatorial objects, where the number of vertices of the flip graph is usually exponential in some parameter, each object has polynomial description length, and a step in the Markov chain can be computed in either polynomial or logarithmic time [16].

Furthermore, a Markov chain is usually called *rapidly mixing* if its *mixing time* is logarithmic in the size of its state space. Thus, even if the USO Markov chain would be rapidly mixing, its mixing time may still be exponential in *n*. A runtime of $O(n\cdot 3^n)$ per step is therefore not the limiting factor for the applicability of the Markov chain in practice.

Finally, we would like to point out that there is a trivial lower bound of $\Omega (2^n)$ for the runtime of a single Markov chain step in the worst case, since it is possible that we may flip all $|E_i|=2^{n-1}$ edges of dimension *i*.

$\textsf{PSPACE}$*-Completeness.* Checking whether any set of edges $S\subseteq E_i$ is a union of phases in a USO *O* is equivalent to checking whether the orientation obtained by flipping *S* is a USO. In the same setting as Theorem 1.1 where an input orientation is given in succinct circuit representation (i.e., by a circuit of size *poly*(*n*) computing the orientation), Gärtner and Thomas [10] showed that recognizing USOs is $\textsf{coNP}$-complete. We thus see that assuming $\textsf{coNP} \ne \textsf{PSPACE}$ the problem of checking whether two edges are in the same phase is strictly harder than the problem of recognizing a USO. Note that our result holds even under the promise that the input is an acyclic USO, a promise that is already $\textsf{PSPACE}$-complete to check on its own [10].

## Preliminaries

The $$n$$*-dimensional hypercube graph* $$Q_n $$ ($$n$$*-cube*) is the undirected graph on the set of vertices $$V(Q_n ) = \{0,1\}^n $$, where two vertices are connected by an edge if they differ in exactly one coordinate. On bitstrings we use $$\oplus $$ for the bit-wise *xor* operation and $$\wedge $$ for the bit-wise *and*. For simplicity we use sets of dimensions and bitstrings interchangeably. For example, for a dimension *i* in the subcube spanned by two vertices $$v, w\in V(Q_n )$$ we write $$i \in v \oplus w$$, instead of having to write $$i \in \{j\;|\;j \in [n], (v\oplus w)_j = 1 \}$$. For a bitstring $$v\in \{0,1\}^n $$ and a number $$i\in [n ]$$, we use the simplified notation $$v\ominus i = v\oplus I_i$$, where $$I_i$$ is the *i*-th standard basis vector. Thus, for a vertex $$v$$ and a dimension $$i\in [n ]$$, the vertex $$v \ominus i$$ is the neighbor of $$v$$ which differs from $$v$$ in coordinate *i*. The edge between $$v$$ and $$v \ominus i$$ is called an *i*-*edge*, or an *edge of dimension* $$i$$. We denote the set of *i*-edges by $$E_i$$.

### Definition 2.1

A *face* of $$Q_n $$ is the induced subgraph corresponding to a subcube. We denote the set of dimensions *spanned* by a face $$f$$ as $$dim(f)$$, and we call $$|dim(f)|$$ the *dimension* of the face. A face of dimension $$n-1$$ is called a *facet*. The facet that spans all dimensions but *i* and contains the vertex $$(0,\ldots ,0)$$ is called the *lower i-facet*. Its opposite facet is called the *upper i-facet*.

An *orientation* *O* is described by a function $O: V(Q_n ) \rightarrow \{0,1\}^n$ such that for all $v\in V(Q_n)$ and all $i\in [n ]$, $O(v)_i\ne O(v\ominus i)_i$. This function assigns each vertex an orientation of its incident edges, called the *outmap* of the vertex, where $O (v)_i=1$ denotes that the *i*-edge incident to vertex $v$ is outgoing from $v$, and $O (v)_i=0$ denotes an incoming edge.

### Definition 2.2

An orientation $$O $$ of $$Q_n $$ is a *unique sink orientation (USO)* if within each face $$f $$ of $$Q_n $$, there exists exactly one vertex with no outgoing edges. That is, there is a unique sink in each face with respect to the orientation $$O$$.

Szabó and Welzl [21] provide a useful characterization for USOs.

### Lemma 2.1

(Szabó-Welzl Condition [21]) An orientation $$O$$ of $$Q_n $$ is a USO if and only if for all pairs of distinct vertices $$v, w \in V(Q_n )$$, we have $$(v \oplus w) \wedge (O (v) \oplus O (w)) \ne 0^n.$$

Bosshard and Gärtner [4] introduced the concept of *pseudo USOs* to capture orientations that are *almost* USOs.

### Definition 2.3

A *pseudo unique sink orientation* (PUSO) of $$Q_n $$ is an orientation $$O$$ that does not have a unique sink, but every proper face of $$Q_n $$ has a unique sink.

Every orientation that is not a USO must contain a minimal face that does not have a unique sink. This minimal face must then be a PUSO. We use this fact in several of our proofs, in conjunction with the following property of PUSOs.

### Lemma 2.2

( [4, Corollary 6 and Lemma 8]) Let $O$ be a PUSO. Then, for every pair of antipodal vertices $v$ and $w$ it holds that $O(v)=O(w)$.

Given an orientation *O* and a set *S* of edges, we write $O\otimes S$ for the orientation *O* with the orientation of all edges in *S* *flipped*, i.e., their orientations are reversed. In general, given a fixed USO *O*, it is quite difficult to characterize which sets *S* of edges lead to $O\otimes S$ being a USO again. In fact, this problem is equivalent to characterizing the set of all USOs. However, the task becomes much easier if we require *S* to consist of only *i*-edges for some dimension *i*, i.e., $S\subseteq E_i$.

Schurr [18] called the minimal sets $S\subseteq E_i$ such that $O\otimes S$ is a USO *i*-*phases.* It turns out that the *i*-phases form a partition of $E_i$. Furthermore Schurr proved that $S\subseteq E_i$ is a union of phases if and only if $O\otimes S$ is also a USO.

The *i*-phases can be characterized as the equivalence classes of the equivalence relation on $E_i$ obtained by taking the transitive closure of the relation of *direct-in-phaseness*.

### Definition 2.4

Let $O$ be an $n$-dimensional USO and $i \in [n ]$ a dimension. Two *i*-edges $e, e' \in E_i$ are *in direct phase* if there exist $v \in e$ and $w \in e'$ such that $O (v)_j = O (w)_j \text { for all } j\in (v\oplus w){\setminus }\{i\}.$

In other words, *e* and $e'$ are in direct phase if two of their incident vertices have the same outmap within the subcube they span, apart from the orientation of the *i*-edges. We can thus see that in this case, *e* and $e'$ must be oriented in the same direction in $O$. Flipping just one of the two edges leads to an immediate violation of the Szabó-Welzl condition by *v* and *w*, the orientations $O \otimes \{e\}$ and $O\otimes \{e'\}$ are not USOs.

**Fig. 1:**
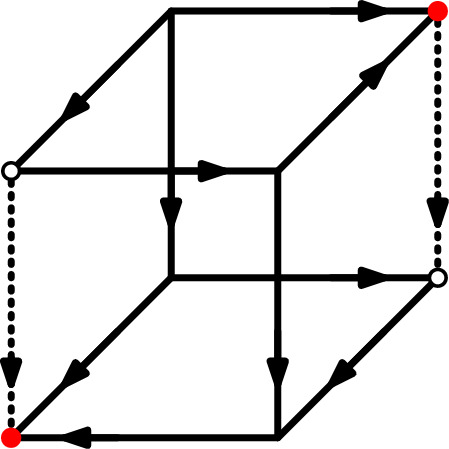
The dotted edges are in direct phase, as certified by the pair of vertices marked in solid red, but not by the vertices circled in black. The phase containing the two dotted edges also contains the front right vertical edge. The back left vertical edge is not in this phase, it is flippable

Further note that *v* and *w* must lie on opposing sides of their respective *i*-edges. However, not both pairs of opposing endpoints of $e$ and $e'$ need to fulfill Definition 2.4. See Fig. 1 for an example of a USO in which two edges are in direct phase but this fact is only certified by one pair of opposing endpoints.

### Definition 2.5

Let *O* be a USO. Two edges $e,e'\in E_i$ are *in phase* if there exists a sequence $e=e_0,e_1,\ldots ,e_{k-1},e_k=e'$ such that for all $j\in [k]$, $e_{j-1}$ and $e_j$ are in direct phase. An *i*-*phase* $P\subseteq E_i$ is a maximal set of edges such that all $e,e'\in P$ are in phase. We write $\mathcal {P}_i(O)$ for the family of all *i*-phases of *O*.

On the one hand, since edges that are in direct phase must be flipped together, we can never flip a set $S\subseteq E_i$ that is not a union of *i*-phases. On the other hand, if flipping some set $S\subseteq E_i$ destroys the Szabó-Welzl condition for some vertices *v*, *w*, their incident *i*-edges must have been in direct phase, and exactly one of the two edges is in *S*. Therefore, flipping a union of *i*-phases always preserves the Szabó-Welzl condition. We thus get the following observation, which we will strengthen in Sect. 3.2.

### Observation 2.1

Let *O* be a USO, and $i\in [n]$ a dimension. For any $S\subseteq E_i$, $O \otimes S$ is a USO if and only if *S* is a union of phases, i.e., $S=\bigcup _{P\in \mathcal {P}'}P$ for $\mathcal {P}'\subseteq \mathcal {P}_i(O)$.

Note that this also implies that flipping a union of *i*-phases does not change the *i*-phases.

A special case is a phase $P=\{e\}$, i.e., a phase that consists of only a single edge. We call such an edge *flippable*. See Fig. 1 again for an example of a flippable edge.

### Lemma 2.3

( [18, Lemma 4.13]) An edge $$\{v,v \ominus i\}$$ in a USO $$O$$ is *flippable* if and only if $$v $$ and $$v \ominus i$$ have the same outmap apart from their connecting *i*-edge, i.e., $$\forall j \in [n ] {\setminus } \{i\}: O (v)_j=O (v \ominus i)_j.$$

## Structural Properties

In this section we show some new structural properties of phases, which are later used to prove algorithmic improvements.

### Connectedness of Phases

As a first question, we investigate whether it is possible that a phase consists of multiple “components” that are far apart. We first show that each edge that is not flippable is in direct phase with at least one “neighboring” edge.

#### Lemma 3.1

Let $e=\{v,v\ominus i\}$ be an *i*-edge. If *e* is not flippable, there exists a dimension $j\ne i$, such that *e* is in direct phase with the edge $\{v\ominus j,(v\ominus i)\ominus j\}$.

#### Proof

Since *e* is not flippable, by Lemma 2.3 there exists at least one dimension $j \in [n ]\setminus \{i\}$ in which some orientation of $v$ and $(v \ominus i)$ disagrees, i.e., $O (v)_j \ne O (v \ominus i)_j$. Thus, in the 2-face spanned by *v* and $(v\ominus i)\ominus j$ the *j*-edges are oriented in the opposite direction. These two vertices certify that *e* and $\{v \ominus j, (v \ominus i)\ominus j\}$ are in direct phase. $\square$

However, edges that are in direct phase with each other are not necessarily neighboring, as can be seen for example in Fig. 1. Nonetheless, we will prove that with respect to this neighboring relation, every phase is *connected*. Let us first define the neighboring relation more formally.

#### Definition 3.1

For some $$i\in [n ]$$ of a cube $$Q_n $$, we say two *i*-edges are *neighboring* when there exists a 2-face containing both of them. Let $$N _i$$ be the graph with $$V(N _i) = E_i$$. There is an edge between two vertices of $$N _i$$ if the vertices correspond to neighboring *i*-edges in $$Q_n $$. We call $$N _i$$ the *neighborhood graph* in dimension *i*.

#### Theorem 3.1

Let $P$ be an *i*-phase of a USO $O$. Then the subgraph of $N_i$ induced by the edges of $P$ is connected.

#### Proof

Let *C* and ${\overline{C}}$ be any non-trivial partition of the edges of *P*. Let $O '$ be the orientation in which we flip all edges in *C* but not the edges in ${\overline{C}}$, i.e., $O ':= O \otimes C$. The orientation $O '$ cannot be a USO, since the edges of *C* and ${\overline{C}}$ are in phase. This means that there is a minimal face *f* such that $O '$ is a PUSO on *f*. It is easy to see that *f* must span dimension *i*. By Lemma 2.2, every antipodal pair of vertices in *f* has the same outmap within the dimensions spanned by *f*, i.e., any such pair of vertices fails the Szabó-Welzl condition. Therefore, within *f*, every pair of antipodal *i*-edges is in direct phase, and when going from the USO *O* to the non-USO $O'$ we must have flipped exactly one edge of the pair. Thus, every such pair consists of exactly one edge in *C* and one edge in ${\overline{C}}$. We can thus see that *f* must contain at least one *i*-edge in *C* that is neighboring an *i*-edge in ${\overline{C}}$. Since this holds for all non-trivial partitions of *P*, the subgraph of $N_i$ induced by *P* must be connected. $\square$

Note that if we would instead consider the subgraph of $N_i$ in which we only use edges $\{e_1,e_2\}$ such that $e_1$ and $e_2$ are both neighboring *and* in direct phase, we could have phases with a disconnected induced subgraph. We give such an example in Sect. 3.3.

### Phases and Matchings

In this section we prove the following generalization of Observation 2.1 from sets $S\subseteq E_i$ to *all* matchings.

#### Theorem 3.2

Let *O* be a USO and $H\subseteq E$ be a matching. Then, $O\otimes H$ is a USO if and only if *H* is a union of phases of *O*.

Schurr already claimed to prove this statement [18, Proposition 4.9]. However, his proof of the “only if” direction contained a severe issue [5]. Let us first restate the “if” direction, which was proven correctly by Schurr.

#### Lemma 3.2

(Theorem 3.2, “if”) Let *O* be a USO and $H\subseteq E$ be a matching that is a union of phases. Then, $O\otimes H$ is a USO.

The “only if” direction we still need to prove can be phrased as follows.

#### Lemma 3.3

(Theorem 3.2, “only if”) Let *O* be a USO and $H\subseteq E$ a matching. Then, if $O\otimes H$ is a USO, *H* is a union of phases of *O*.

Schurr claimed to prove Lemma 3.3 by contraposition: Assume *H* is not a union of phases. Then, there must be a phase *P* and two edges $e,e'\in P$ such that $e\in H$ and $e'\not \in H$. While *e* and $e'$ are not necessarily directly in phase, there must be a sequence of direct-in-phaseness relations starting at *e* and ending in $e'$. At some point in this sequence, there must be two edges $e_i\in H$ and $e_{i+1}\not \in H$ that are in direct phase. Schurr then argues that since we flip only $e_i$ but not $e_{i+1}$, the vertices $v\in e_i$ and $w\in e_{i+1}$ certifying that these edges are in direct phase must violate the Szabó-Welzl condition after the flip. Thus, $O\otimes H$ would not be a USO.

However, there is a crucial issue in this proof: Since we are assuming that $e'\not \in H$, it is possible that there is some edge $f\in H\cap E_j$ for some $j\ne i$, such that *w* is incident to *f*. Flipping this edge *f* as well may prevent *v* and *w* from violating the Szabó-Welzl condition.

This issue seems very difficult to fix, since the core of the argument (the outmap of *w* not being changed) is wrong. We therefore prove Lemma 3.3 in a completely different way than Schurr, for which we first need the following observation.

#### Observation 3.1

Let $O$ be a USO and $H$ a union of phases in $O$. Let $P$ be a set of *i*-edges such that $H \cap P =\emptyset$ and $H \cup P$ is a matching. If $P$ is a phase in $O$, it is a union of phases in $O \otimes H$.

#### Proof

If $P$ is a phase in $O$, by Lemma 3.2, both $O$ with $H$ flipped, and $O$ with $H \cup P$ flipped are USOs. Their difference is $P$, and thus by Observation 2.1, $P$ is a union of phases in $O \otimes H$. $\square$

We prove Lemma 3.3 with the help of the following *minimization* lemma. Note that a *counterexample to Lemma* 3.3 is a pair (*O*, *H*) such that *O* is a USO and *H* is a matching that is not a union of phases in *O*, but $O\otimes H$ is a USO nonetheless.

#### Lemma 3.4

(Minimization Lemma) If there exists a counterexample to Lemma 3.3, then there also exists a counterexample $(O,H)$ such that for each facet $F$ of $O$, $H \cap F$ is a union of phases in $F$, and such that $H$ contains no phase of $O$.

We prove this lemma in turn with another, weaker, minimization lemma.

#### Lemma 3.5

(Weak Minimization Lemma) If there exists a counterexample $(O ^*,H ^*)$ to Lemma 3.3, then there also exists a counterexample $(O,H)$ with $dim(O ^*)=dim(O)$ in which $H$ does *not* contain a whole phase of $O$.

#### Proof

Let *U* be the union of all phases $$P$$ of $$O ^*$$ that are fully contained in the matching, i.e., $$P \subseteq H ^*$$. By Lemma 3.2, $$O:=O ^*\otimes U$$ is a USO. We denote by $$H $$ the set $$H ^*\setminus U$$, which is a matching containing only incomplete sets of phases of $$O ^*$$.

We now argue that $$(O,H)$$ is a counterexample with the desired property. As $$H ^*$$ originally was not a union of phases, $$H \not =\emptyset $$. Furthermore, $$O \otimes H $$ is equal to $$O ^*\otimes H ^*$$ and thus by assumption a USO. It remains to be proven that $$H $$ is not a union of phases of $$O $$, and in particular contains no phase completely.

To do so, we first prove that *U* is a union of phases in $$O $$. One can see this by successively flipping in $$O ^*$$ the sets $$U_i:=U\cap E_i$$ which decompose *U* into the edges of different dimensions. After flipping each set $$U_{i}$$, by Observation 3.1 all the other sets $$U_{j}$$ remain unions of phases. Furthermore, as flipping a union of *i*-phases does not change the set of *i*-phases, $$U_{i}$$ also remains a union of phases.

Now, by Observation 3.1, any phase $$P \subseteq H $$ of $$O $$ is a union of phases in $$O \otimes U = O ^*$$. But then, by definition of *U*, *P* would have been included in *U*. Thus, we conclude that $$H $$ does not contain any phase of $$O $$. $$\square $$

Let us now use Lemma 3.5 to prove Lemma 3.4.

#### Proof of Lemma 3.4

Let $$(O,H)$$ be a smallest-dimensional counterexample to Lemma 3.3 among all counterexamples $$(O ^*,H ^*)$$ where $$H ^*$$ contains no phase of $$O ^*$$. By Lemma 3.5, at least one such counterexample must exist, thus $$(O,H)$$ is well-defined. Let *n* be the dimension of $$O $$.

We now prove that $H\cap F$ is a union of phases in *F* for all facets *F*: If this would not be the case for some *F*, then constraining $O$ and $H$ to $F$ would yield a counterexample $(O _F,H _F)$ of Lemma 3.3 of dimension $n-1$. By applying Lemma 3.5, this counterexample can also be turned into a $(n-1)$-dimensional counterexample $(O _F ',H _F ')$ such that $H _F '$ contains no phase of $O _F '$. This is a contradiction to the definition of $(O,H)$ as the smallest-dimensional counterexample with this property. We conclude that $(O,H)$ is a counterexample with the desired properties. $\square$

With this lemma we are ready to prove Lemma 3.3, and thus also Theorem 3.2.

#### of Lemma 3.3

By Lemma 3.4 it suffices to show that there exists no counterexample $(O,H)$ to this lemma such that $H$ contains no phase of $O$ and in each facet $F$ of $O$, $H \cap F$ is a union of phases.

Assume that such a counterexample $(O,H)$ exists. For each dimension $i \in [n ]$ for which $H _i:=H \cap E_i$ is non-empty we consider the orientation $O _i:= O \otimes H _i$. By assumption, $H _i$ is not a union of phases of $O$, and thus $O _i$ is not a USO. Furthermore, as $H _i$ is a union of phases in each facet of $O$, all facets of $O _i$ are USOs. Thus, $O _i$ is a PUSO. Recall that by Lemma 2.2, in a PUSO, every pair of antipodal vertices has the same outmap. For $O _i$ to be a PUSO, and $O$ to be a USO, exactly one vertex of each antipodal pair of vertices must be incident to an edge in $H _i$.

By Observation 2.1, if (*O*, *H*) is a counterexample, we know that $$H$$ must contain edges of at least two dimensions, *i* and *j*. Consider $$H _i$$ and $$H _j$$. By the aforementioned argument, both $$O _i$$ and $$O _j$$ are PUSO. As $$H$$ is a matching, there is no vertex incident to both an edge of $$H _i$$ and $$H _j$$. Therefore, for each pair of antipodal vertices $$v, w $$, one is incident to an edge of $$H _i$$, and one to an edge of $$H _j$$. Since $$v$$ and $$w$$ must have the same outmaps in both $$O _i$$ and $$O _j$$, we get the following two conditions:

1. $$(v \oplus w) \wedge (O (v) \oplus O (w)) = I_i,$$ and
2. $$(v \oplus w) \wedge (O (v) \oplus O (w)) = I_j.$$

Clearly, this implies $I_i=I_j$ and we have thus obtained a contradiction, and no counterexample can exist to Lemma 3.3. $\square$

Now that we have recovered Theorem 3.2, we can also strengthen Observation 3.1.

#### Theorem 3.3

Let $O$ be a USO and $H$ a union of phases in $O$. Let $P$ be a set of *i*-edges such that $H \cap P =\emptyset$ and $H \cup P$ is a matching. Then, $P$ is a phase in $O$ if and only if it is a phase in $O ':= O \otimes H$.

#### Proof

We first prove that *P* is a *union of* phases in $O$ if and only if it is a *union of* phases in $O '$. By Theorem 3.2, $H$ is a union of phases in both $O$ and $O '$. Thus, this follows from Observation 3.1.

Now, assume for a contradiction that *P* is a single phase in $O$ but a union of multiple phases in $O '$ (the other case can be proven symmetrically): Then, let $P'\subset P$ be a phase of $O '$. By the statement proven above, $P'$ is a union of phases in $O$. However, $P'\subset P$ and *P* is a single phase. This yields a contradiction, and thus the lemma follows. $\square$

We thus conclude that every phase remains a phase when flipping some matching that does not share any vertex with the phase.

### Large-Distance Dependencies: The *n*-Schurr Cube

When trying to find an efficient algorithm for computing phases, one might ask the following: Is there some small integer *k*(*n*), such that for every *n*-dimensional USO, the transitive closure of the direct-in-phaseness relation stays the same when only considering the relation between pairs of *i*-edges which have a distance of at most *k*(*n*) to each other in $N_i$? In other words, does it suffice to compute the direct-in-phaseness relationships only for edges that are close to each other, instead of for all pairs of *i*-edges?

**Fig. 2:**
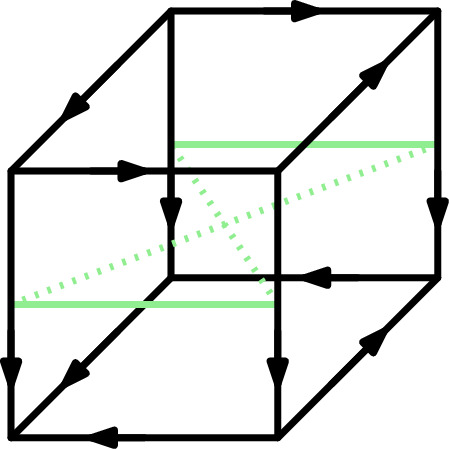
The 3-Schurr Cube. The vertical edges are all in phase. The direct-in-phase relationships between these edges are marked in green. Note that when disregarding the direct-in-phase relationships of antipodal edges (dotted), this phase splits in two parts

In this section we will show that for every *n* there exists an *n*-dimensional USO in which a direct-in-phase relationship between some antipodal *i*-edges is necessary to define some *i*-phase, i.e., we show that no such $k(n)<n-1$ exists. Such a USO was found by Schurr [18] for $n=3$ and is shown in Fig. 2: All four vertical edges are in phase, but if only direct-in-phaseness between non-antipodal edges is considered, there would be no connection between the front and the back facet.

We generalize the properties of this 3-Schurr cube to the $n$-Schurr cube, i.e., we show that there exists an *n*-dimensional USO such that

- all *n*-edges are in phase,
- all *n*-edges are in direct phase with their antipodal *n*-edge, and
- if ignoring the $(n-1)$-direct-in-phaseness, the phase splits apart.

We can obtain a cube fulfilling this in a recursive fashion:

#### Definition 3.2

(The *n*-Schurr cube) Let $S^1$ be the 1-dimensional USO consisting of a single edge oriented towards 0. For $n\ge 2$, let $S^n$ be the cube obtained by placing $S^{n-1}$ in the lower *n*-facet, and $S^{n-1}\otimes E_{n-1}$ in the upper *n*-facet, with all *n*-edges oriented towards the lower *n*-facet.

Alternatively, we can define the same cube without recursion, simplifying its analysis:

$$\begin{aligned}\forall v\in \{0,1\}^n:\; S^n(v)_i={\left\{ \begin{array}{ll} v_i\oplus v_{i+1} &  \text { for } i<n,\\ v_n &  \text { for } i=n. \end{array}\right. }\end{aligned}$$

**Fig. 3:**
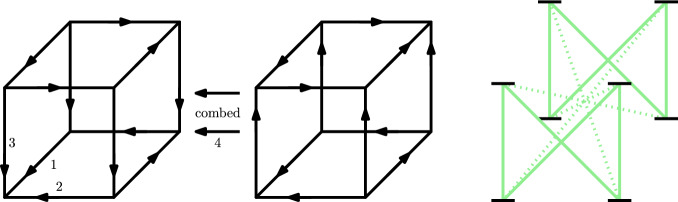
The 4-Schurr Cube. The combed edges between the two pictured 4-facets are in one phase. The direct-in-phase relationships between these edges is shown on the right. Note that when disregarding the direct-in-phase relationships of antipodal edges (dotted), the phase splits in two parts

An example of this cube for $n=4$ can be seen in Fig. 3. This cube fulfills the properties outlined above:

#### Theorem 3.4

In $S^n$ as defined in Definition 3.2, all *n*-edges are in direct phase with the antipodal edge (certified by both pairs of antipodal endpoints), and all *n*-edges are in phase. No *n*-edge is in direct phase with any non-antipodal *n*-edge located in the opposite 1-facet.

#### Proof

We first see that every pair *v*, *w* of antipodal vertices certifies their incident *n*-edges to be in direct phase, since for all $i<n$, $v_i\oplus v_{i+1}=w_i\oplus w_{i+1}$ and thus $S^n(v)_i=S^n(w)_i$.

Next, we show that no *n*-edge is in direct phase with a non-antipodal *n*-edge in the opposite 1-facet. Let *v* be any vertex and *w* a vertex such that $w_{1}\ne v_{1}$, $w_n\ne v_n$, but $v_i=w_i$ for some *i*. Let $i'$ be the minimum among all *i* with $v_i=w_i$. Note that $i'>1$. Then, we have $v_{i'-1}\ne w_{i'-1}$, but we also have $S^n(v)_{i'-1}=v_{i'-1}\oplus v_{i'} \ne w_{i'-1}\oplus w_{i'}=S^n(w)_{i'-1}$, and thus the *n*-edges incident to *v* and *w* are not in direct phase.

By a similar argument one can see that two vertices *v* and *w* certify their incident *n*-edges to be in direct phase if and only if there exists some integer $1<k\le n$ such that $v_i=w_i$ for all $i<k$, and $v_i\ne w_i$ for all $i\ge k$. From this, it is easy to see that all *n*-edges are in phase: The *n*-edges in the upper 1-facet are each in direct phase with some edge in the lower 1-facet. The lower 1-facet is structured in the same way as the cube $S^{n-1}$, thus we can inductively see that all *n*-edges in this facet are in phase. Therefore, all *n*-edges of $S^n$ are in phase. $\square$

### Phases and Hypervertices

A face $$f$$ of a cube $$Q_n $$ is called a *hypervertex*, if and only if for all vertices $$v, w \in f $$ and all dimensions *i* not spanned by *f*, we have $$O (v)_i = O (w)_i$$. In other words, for each dimension *i*, all *i*-edges between *f* and the rest of the cube are oriented the same way.

By Lemma 2.3, a hypervertex of dimension 1 is thus a flippable edge. We therefore know that we can change the orientation within a one-dimensional hypervertex arbitrarily without destroying the USO condition. The following lemma due to Schurr and Szabó generalizes this to higher-dimensional hypervertices.

#### Lemma 3.6

([19, Corollary 6]) Let *O* be a *n*-dimensional USO, and $$f$$ some *k*-dimensional hypervertex. Then, the orientation on the face $$f$$ can be replaced by any other *k*-dimensional USO $$O '_f $$, such that the resulting orientation $$O '$$ is also a USO:

$$\begin{aligned}\forall v\in V(Q_n ),i\in [n ]: O '(v)_i:= {\left\{ \begin{array}{ll} O (v)_i &  \text {if }f _i\not =* \text { or } v\not \in f, \\ O '_f (\{v_j \;|\; f _j = *\})_i &  \text {otherwise}. \end{array}\right. } \end{aligned}$$

Hypervertices thus allow for a local change of the orientation. We will now investigate what this implies about the structure of phases. In particular, we prove the following two statements, given an *n*-dimensional USO *O* and some face $f$.

#### Theorem 3.5

The face *f* is a hypervertex if and only if for all $i\in dim(f)$, $e \in E(f)_i$ and $e' \in E(Q_n {\setminus } f)_i$, $e$ and $e'$ are not in phase.

#### Theorem 3.6

If there exists some $i \in dim(f)$ such that $E(f)_i$ is an *i*-phase of *O*, then $f$ is a hypervertex.

In other words, Lemma 3.5 says that a face is a hypervertex if and only if for every edge $e$ of $f$, the phase containing $e$ is contained within $f$. Theorem 3.6 gives a slightly different sufficient condition for *f* being a hypervertex: if *all* edges of *one* dimension of $f$ are exactly a *single* phase, $f$ is a hypervertex.

#### of Lemma 3.5

We first prove the “if” direction. Assume $f$ is not a hypervertex. Then $f$ has at least one incoming and one outgoing edge in some dimension $j \notin dim(f)$. More specifically there exists a 2-face crossing between *f* and $Q_n{\setminus } f$ that has the aforementioned incoming and outgoing edge, i.e., a 2-face with $\{v, w \} \in E(f)_i$, $\{(v \ominus j), (w \ominus j)\} \in E(Q_n {\setminus } f)_i$ and $O (v)_j \ne O (w)_j$. This however implies that the edge $\{v, w \}$ is in direct phase with the edge $\{(v \ominus j), (w \ominus j)\}$.

Next, we prove the “only if” direction and assume $f$ is a hypervertex. Let $i \in dim(f)$, $e \in E(f)_i$ and $e' \in E(Q_n \setminus f)_i$. By Lemma 3.6 we can change the current orientation $O_f$ of a hypervertex $f$ to any arbitrary USO of the same dimension. We can thus replace it by the USO $O_f'=O_f\otimes E(f)_i$, i.e., $O_f$ with all edges of dimension *i* flipped. This flips $e$ but not $e'$, and the result is still USO. Thus, by Observation 2.1, $e$ and $e'$ are not in phase. $\square$

To prove Theorem 3.6 we use the connectedness of phases (Theorem 3.1) as well as the *partial swap* construction of Borzechowski, Doolittle, and Weber [2]. For any dimension *i*, the partial swap construction considers the set *S* of *i*-edges that are oriented upwards. Then, the subgraphs of the upper *i*-facet and the lower *i*-facet induced by vertices incident to *S* are *swapped*. Note that these subgraphs are not necessarily subcubes.

#### of Theorem 3.6

We run a proof by contradiction. Assume *O* is a USO with a dimension $i \in dim(f)$ such that $E(f)_i$ is an *i*-phase of *O*, but $f$ is *not* a hypervertex. Thus, there exists a dimension $j \in [n] \setminus dim(f)$ such that for some pair of vertices $v, w \in f$, the orientations of the connecting edges $\{v, v \ominus j \}$ and $\{w, w \ominus j \}$ differ.

First, we switch our focus to the $n'$-dimensional face $f'$ that contains *f* and for which $dim(f')=dim(f)\cup \{j\}$. Without loss of generality, we assume *f* is the lower *j*-facet of $f'$. Second, we adjust the orientation of the *i*-edges. We let all *i*-edges in $E(f)$ point upwards and all *i*-edges in $E(f') {\setminus } E(f)$ point downwards. Since $E(f)_i$ is a phase of *O*, $E(f')_i\setminus E(f)_i$ is a union of phases of *O* restricted to $f'$, and thus the resulting orientation $O'$ of $f'$ is a USO.

For all $\{v, w\} \in E(f)_i$ it holds that $O(v)_j = O(w)_j$, since otherwise the edge $\{v,w\}$ would be in direct phase with the edge $\{v\ominus j,w\ominus j\}$. Thus, the orientation of the *j*-edges splits $E(f)_i$ into two parts:

$$\begin{aligned} E(f)_i^+&:= \{ \{v, w\} \in E(f)_i \;|\; O'(v)_j = O'(w)_j=0 \} \text { and} \\ E(f)_i^-&:= \{ \{v, w\} \in E(f)_i \;|\; O'(v)_j = O'(w)_j = 1 \}. \end{aligned}$$

**Fig. 4:**
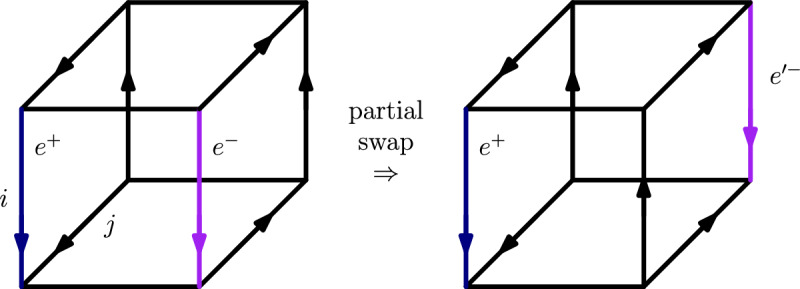
Sketch of $O'$ and $O''$ from the proof of Theorem 3.6. The edges $e^+\in E(f)_i^+$ and $e^-\in E(f)_i^-$ are pulled apart by the partial swap. If they were in direct phase before they must still be in direct phase, however the phase connecting $e^+$ and $e'^-$ after the partial swap cannot be connected

As $E(f)_i$ is a phase, there must be some edge $e ^+\in E(f)_i^+$ which is in direct phase to some edge $e ^-\in E(f)_i^-$. We now perform a partial swap (see [2]) on $O '$ in dimension *j*, yielding a USO $O ''$. See Fig. 4 for a sketch of $O'$ and $O''$. The endpoints of $e ^+$ are not impacted by this operation, but the endpoints of $e ^-$ are moved to the opposite *j*-facet, now forming a new edge ${e '}^{-}$. The two edges $e^+$ and ${e '}^{-}$ must be in direct phase in $O''$, since in dimension *j* all four of their endpoints are incident to an incoming edge, and in all the other dimensions the outmaps of the endpoints of ${e '}^{-}$ in $O''$ are the same as the outmaps of the endpoints of ${e}^-$ in $O'$.

For every pair of *i*-edges neighboring in dimension *j*, exactly one is upwards and one is downwards oriented. Let *P* be the phase containing $e ^+$ and ${e '}^-$. Since *P* contains only upwards oriented *i*-edges, and since *P* contains at least one edge of each *j*-facet, the subgraph of $N _i$ induced by *P* cannot be connected. This is a contradiction to Theorem 3.1, which proves the lemma. $\square$

The proof above actually also implies a version of Theorem 3.6 for phases that are not full faces.

#### Theorem 3.7

Let *O* be a USO on the cube $$Q_n $$ and *P* an *i*-phase of *O*. Let $$f$$ be the minimal face with $$P \subseteq E(f)_i$$. Then, for all dimensions $$j \in [n] {\setminus } dim(f) $$ and for all $$v, w \in P$$, we have $$O(v)_j = O(w)_j$$.

## Algorithms to Compute Phases

The definitions of direct-in-phaseness and phases (Definitions 2.4 and 2.5) naturally imply a simple algorithm to compute all phases of a USO: Compare every pair of vertices and record the edges that are in direct phase, and then run a connected components algorithm on the graph induced by these direct-in-phase relationships. This takes $O(4^n)$ vertex comparisons (and thus $O(n\cdot 4^n)$ time) for an *n*-dimensional cube. As we will see, we can do better.

Based on Observation 2.1 we get a natural connection between USO recognition and computation of phases; if *O* is a USO and $H\subseteq E_i$ a set of *i*-edges, then *H* is a union of phases if and only if $O\otimes H$ is a USO. However, naively applying a USO recognition to exploit this would be highly inefficient for computing phases, since we would have to check whether $O\otimes H$ is a USO for many different candidate sets *H*. We will see that a procedure that is very similar to just a single run of a USO recognition algorithm actually suffices to be able to compute all phases.

To achieve this, we can profit from the similarity of the Szabó-Welzl condition (Lemma 2.1) and the condition for being in direct phase (Definition 2.4). Let $\mathcal {A}$ be an algorithm for USO recognition that works by testing the Szabó-Welzl condition for some fixed (i.e., input-independent) subset *T* of all vertex pairs $$\left( {\begin{array}{c}V(Q_n)\\ 2\end{array}}\right) $$, and outputs that the given orientation is a USO if and only if all pairs in *T* fulfill the Szabó-Welzl condition. We will show that then there exists an algorithm $\mathcal {B}$ for computing all phases of a given USO that also only compares the vertex pairs *T*. Our phase computation algorithm $\mathcal {B}$ is based on the following symmetric relation.

### Definition 4.1

Let $$T\subseteq \left( {\begin{array}{c}V(Q_n)\\ 2\end{array}}\right) $$. Two *i*-edges $e,e'$ are *in direct T-phase*, if

- *e* and $e'$ are in direct phase and
- there exist $v \in e$, $v' \in e'$ such that $v,v'$ certify $e,e'$ to be in direct phase, and $\{v,v'\}\in T$.

### Lemma 4.1

Let $$T\subseteq \left( {\begin{array}{c}V(Q_n)\\ 2\end{array}}\right) $$ be the set of vertex pairs compared by a Szabó-Welzl condition-based USO recognition algorithm $\mathcal {A}$. In every USO *O*, the transitive closure of direct-in-*T*-phaseness is equal to the transitive closure of direct-in-phaseness.

### Proof

Clearly, by definition, the equivalence classes of direct-in-*T*-phaseness (called *T*-*phases*) are a refinement of phases. We now show that every *T*-phase is also a phase. Assume there is a *T*-phase *B* (in dimension *i*) that is a proper subset of a phase *P*. Then, by Observation 2.1, $O\otimes B$ is not a USO. Algorithm $\mathcal {A}$ must be able to detect this. Thus, there is a vertex pair $\{v,v'\}\in T$ that violates the Szabó-Welzl condition in $O\otimes B$. Clearly, exactly one of the *i*-edges incident to $v,v'$ is contained in *B*. Since flipping exactly one of these edges led to $v,v'$ violating the Szabó-Welzl condition, these edges are in direct phase in *O*, and thus they are also in direct *T*-phase. We conclude that both edges should be in the same *T*-phase, and thus have a contradiction. $\square$

With this lemma, we can turn the USO recognition algorithm $\mathcal {A}$ into a phase computation algorithm $\mathcal {B}$: For each pair of vertices in *T*, find the edges they certify to be in direct *T*-phase; then, calculate the connected components. By Lemma 4.1 this yields the phases of *O*.

The best known USO recognition algorithm—the one based on the work of Bosshard and Gärtner on PUSOs [4]—uses a set *T* of size $|T|=3^n$: this follows from the fact that Lemma 2.2 implies that for each face, only the minimum and maximum vertices need to be compared. Thus, we can also calculate the phases with $O(3^n)$ vertex comparisons and thus $O(n\cdot 3^n)$ time. Note that any better Szabó-Welzl condition-based USO recognition algorithm discovered in the future would also imply further improvements in phase computation.

We end this section by showing that the phase containing a given edge *e* can be flipped in polynomial space.

### Lemma 4.2

Let *O* be a USO of $$Q_n $$ given by an oracle allowing for read and write access to single edges. Let $$e\in E_i$$ be a given edge. Then, there exists an algorithm to flip the unique phase containing *e* using *poly*(*n*) space.

### Proof

To flip the phase containing the *i*-edge *e* we iterate through each edge $e'\in E_i$ and check whether $e'$ and *e* are in phase, i.e., whether $e'$ should be flipped. To check this, we first provide a nondeterministic algorithm: we start at the edge *e* and iteratively guess the next edge that is in direct phase with the previous edge. If in this way we can reach $e'$ in at most $2^{n-1}$ steps, *e* and $e'$ must be in phase. Such an algorithm only needs *O*(*n*) bits to store the current and the next guessed edge of the sequence, and *O*(*n*) bits to store the current length of the path.

By Savitch’s theorem [17], $\textsf{NPSPACE} =\textsf{PSPACE}$, and thus the algorithm above can be turned into a deterministic algorithm using only polynomial space as well. $\square$

## $\textsf{PSPACE}$-Completeness

Checking whether two edges are in *direct* phase in a USO is trivial: It can be achieved with just four evaluations of the outmap function and *O*(*n*) additional time. In this section we prove that testing whether two edges are in phase (not necessarily directly) is $\textsf{PSPACE}$-complete, i.e., we prove Theorem 1.1. We first have to make the computational model clearer: Since a USO is a graph of exponential size (in the dimension *n*), the usual way of specifying a USO is by a *succinct* representation, i.e., a Boolean circuit computing the outmap function with *n* inputs and *n* outputs and overall size polynomial in *n*. This reflects the practical situation very well, since in all current applications of USO sink-finding, the outmap function can be evaluated in time polynomial in *n*.

### Definition 5.1

The decision problem 2InPhase is to decide the following question. Given an *n*-dimensional USO $$O$$ by a Boolean circuit of size in *O*(*poly*(*n*)) and two *i*-edges $$e, e' $$, are $$e$$ and $$e'$$ in phase?

Let us restate Theorem 1.1 using this new definition.

### Theorem 5.1

2InPhase is $\textsf{PSPACE}$-complete, even when the input is guaranteed to be an acyclic USO and *e* and $e'$ are antipodal.

Before proving Theorem 5.1, we would like to point out the following problem which inherits $\textsf{PSPACE}$-hardness from 2InPhase.

### Definition 5.2

The decision problem USOCompletion is to decide the following question:

Given a partial orientation $O$ of an *n*-dimensional hypercube by a Boolean circuit of size in *O*(*poly*(*n*)) computing $O:V(Q_n)\rightarrow \{0,1,-\}^n$ (where 0 and 1 denote incoming and outgoing edges, and − denotes an unoriented edge), is $O$ extendable to a USO?

We now show that 2InPhase reduces to USOCompletion. Unlike 2InPhase, we do not know whether USOCompletion is in $\textsf{PSPACE}$.

### Corollary 5.1

USOCompletion is $\textsf{PSPACE}$-hard.

### Proof

We reduce 2InPhase to USOCompletion: We change the circuit such that in dimension *i*, all edges become unoriented, except *e* and $e'$, which are oriented in opposite directions. Clearly, if this partial orientation is extendable to a USO, *e* and $e'$ cannot be in phase since they would have to be oriented in the same direction, which they are not. If the partial orientation is not extendable, *e* and $e'$ must be directed in the same way in all possible connections of the two *i*-facets, i.e., they must be in the same *i*-phase. $\square$

We finally prove Theorem 5.1.

### Proof of Theorem 5.1

We already showed that 2InPhase can be solved in polynomial space implicitly in the proof of Lemma 4.2. It remains to show that 2InPhase is $\textsf{PSPACE}$-hard. Since our reduction will only generate acyclic USOs, the problem remains $\textsf{PSPACE}$-hard even under the promise that the input function specifies an acyclic USO. For our proof we reduce the following (standard) $\textsf{PSPACE}$-complete problem.

### Definition 5.3

*Quantified Boolean Formula (QBF)* asks to decide the following: Given a formula $\Phi$ in conjunctive normal form on the variables $x_1,\ldots ,x_n$, as well as a set of quantifiers $q_1,\ldots ,q_n\in \{\exists ,\forall \}$, decide whether the sentence $q_1x_1,\ldots ,q_nx_n:\Phi (x_1,\ldots ,x_n)$ is true.

We reduce QBF to 2InPhase. To prove $\textsf{PSPACE}$-hardness, this reduction must be polynomial-time and many-one. We translate $q_1x_1,\ldots ,q_nx_n:\Phi (x_1,\ldots ,x_n)$ into an acyclic USO $O_0[]$, built recursively from the USOs $O_{1}[0]$ and $O_{1}[1]$ which correspond to the sentences $q_2x_2,\ldots ,q_{n}x_{n}:\Phi (0,x_2,\ldots ,x_{n})$ and $q_2x_2,\ldots ,q_{n}x_{n}:\Phi (1,x_2,\ldots ,x_{n})$, respectively. In general, we let a USO $O_i[b_{1},\ldots ,b_{i}]$ for $b_j\in \{0,1\}$ correspond to the sentence $q_{i+1}x_{i+1},\ldots ,q_nx_n:\Phi (b_{1},\ldots ,b_i,x_{i+1},\ldots ,x_n)$.

We show inductively that all of our orientations $O_i[b_{1},\ldots ,b_i]$ fulfill the following invariants.

- $$O_i[b_{1},\ldots ,b_i]$$ is a USO.
- $$O_i[b_{1},\ldots ,b_i]$$ is acyclic.
- $O_i[b_{1},\ldots ,b_i]$ is combed downwards in dimension 1, i.e., all 1-edges point towards the lower 1-facet.
- The minimum vertex of $O_i[b_{1},\ldots ,b_i]$ is its sink, the maximum vertex is its source.
- In $O_i[b_{1},\ldots ,b_i]$, the 1-edges incident to the minimum and maximum vertices are in phase if and only if the sentence
  $$\begin{aligned} q_{i+1}x_{i+1},\ldots ,q_nx_n:\Phi (b_{1},\ldots ,b_i,x_{i+1},\ldots ,x_n) \end{aligned}$$
  is true.

If we can show these properties, the only step left is to show that a circuit computing $O_0[]$ can be constructed in polynomial time.

**Fig. 5:**
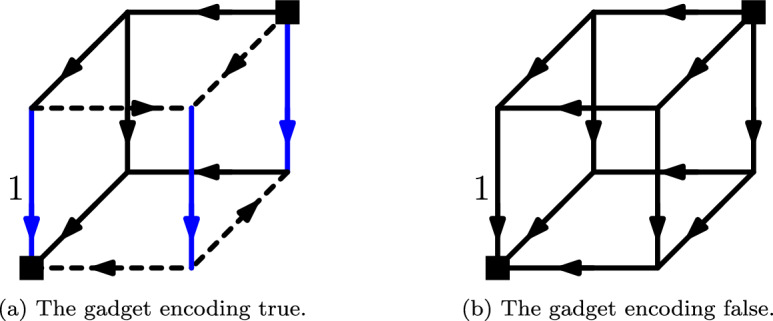
The two base gadgets. In the true-gadget the blue vertical 1-edges are in phase, in particular those incident to the minimum and maximum vertices (square). In the false-gadget these two edges are flippable and thus not in phase

We first begin by discussing the anchor of our recursive construction, namely $O_n$: The orientations $O_n[b_1,\ldots ,b_n]$ correspond to the (unquantified) sentences $\Phi (b_1,\ldots ,b_n)$. The truth value of such an unquantified sentence can be efficiently tested (one simply needs to evaluate $\Phi$ once), and we can thus set these orientations to be one of two fixed orientations: the *true-* or the *false-gadget*, as seen in Fig. 5. Let us now show that these two gadgets fulfill the invariants.

*The Base Gadgets* The two base gadgets, the true- and the false-gadget, are the 3-dimensional USOs shown in Fig. 5. As can be seen, they are both acyclic USOs with sink and source at the minimum and maximum vertex, respectively, and combed downwards in dimension 1. In the true-gadget, the minimum and maximum vertex of each 1-facet are connected by a path of two edges (dashed) whose orientations are different in the upper and lower 1-facets. Thus, along this path, the incident 1-edges are always in direct phase. We can thus see that the 1-edges incident to the minimum and maximum vertices must be in phase, as required. In contrast, in the false-gadget every 1-edge is flippable (since the gadget is just a uniform USO), and thus the 1-edges incident to the minimum and maximum vertices are not in phase. We thus conclude that the base cases of our induction hold.

*The* $\forall$ *Quantifier* We now show how we build a USO $\mathcal {O}:=O_i[b_{1},\ldots ,b_i]$, if $q_{i+1}=\forall$. We first note that $q_{i+1}x_{i+1},\ldots ,q_nx_n:\Phi (b_{1},\ldots ,b_i,x_{i+1},\ldots ,x_n)$ is true if and only if *both* of the sentences

$$\begin{aligned} q_{i+2}x_{i+2},\ldots ,q_nx_n:\Phi (b_{1},\ldots ,b_i,B,x_{i+2},\ldots ,x_n) \end{aligned}$$

for $B\in \{0,1\}$, i.e., the two sentences corresponding to $\mathcal {F}:=O_{i+1}[b_{1},\ldots ,b_i,0]$ and $\mathcal {T}:=O_{i+1}[b_{1},\ldots ,b_i,1]$, are true.

**Fig. 6:**
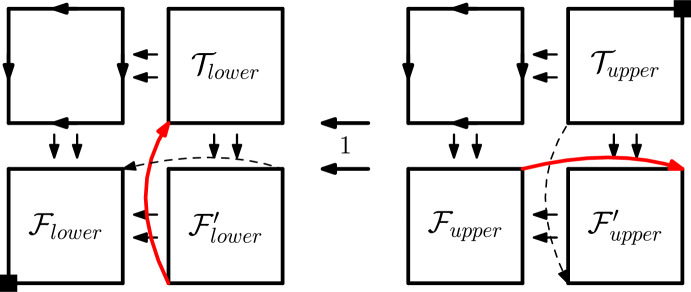
The $\forall$ construction. The 1-edges incident to the square vertices are only in phase if the minimum and maximum 1-edges of both $\mathcal {F}$ and $\mathcal {T}$ are in phase

We show how to build $\mathcal {O}$ from $\mathcal {T}$ and $\mathcal {F}$ in Fig. 6. Essentially, $\mathcal {O}$ consists of two copies of $\mathcal {F}$ ($\mathcal {F}$ and $\mathcal {F}'$), one copy of $\mathcal {T}$, and one uniform USO, all connected in a combed way, but then two specially marked red edges are flipped. These red edges are the edge connecting the minimum vertices of $\mathcal {F'}$ and $\mathcal {T}$, and the edge connecting the maximum vertices of $\mathcal {F}$ and $\mathcal {F'}$. Note that by the inductive hypothesis, we can assume both $\mathcal {F}$ and $\mathcal {T}$ to fulfill the invariants outlined above. We need to prove $\mathcal {O}$ must fulfill the invariants too.

We first prove that $\mathcal {O}$ is a USO: Since all four “ingredients” are USOs, before flipping the red edges the orientation is clearly a USO (it can be seen as a product construction as described in [19]). We thus only have to show that the two red edges are flippable. The red edge in the top 1-facet goes between the maximum vertices of $\mathcal {F}_{upper}$ and $\mathcal {F}'_{upper}$, which by inductive hypothesis are both sources of their respective subcubes. Thus, the two endpoints have the same outmap, and this red edge is flippable. The same argument works for the red edge in the bottom 1-facet, which goes between two sinks. Thus, the orientation is a USO.

Next, we prove that the construction preserves acyclicity: We can view both 1-facets independently, since the 1-edges are combed and thus cannot be part of a cycle. In a similar way, we can split each 1-facet further along some combed dimension. In the resulting subcubes, since the uniform USO, $\mathcal {F}$, and $\mathcal {T}$ are acyclic, any cycle must use one of the red edges. However, in these subcubes each red edge either ends at a sink or starts at a source, and can thus not be part of any directed cycle. Thus, $\mathcal {O}$ is acyclic.

Next, we see that $\mathcal {O}$ is combed downwards in dimension 1, and since the minimum vertex of $\mathcal {F}$ is a sink and the maximum vertex of $\mathcal {T}$ is a source, the sink and source of $\mathcal {O}$ are also located at the minimum and maximum vertex, respectively.

Finally, we need to show that the 1-phases of $\mathcal {O}$ are correct. In other words, we wish to prove that the 1-edges incident to the minimum and maximum vertices are in phase if and only if this holds for *both* $\mathcal {F}$ and $\mathcal {T}$. The “if” direction is easy to see, since we have a chain of direct in-phaseness: We can first go through $\mathcal {F}$, then cross over to the right (since the red and dashed edges go in the opposite direction, their incident 1-edges are in phase), take the same path back through $\mathcal {F}'$, cross upwards along the red and dashed edges, and finally go through $\mathcal {T}$. For the “only if” direction, we can assume that the 1-edges incident to the minimum and maximum vertices are not in phase in either $\mathcal {F}$ or $\mathcal {T}$. Thus, there must be some phase *P* in $\mathcal {F}$ that includes the 1-edge incident to its source but not the one incident to its sink, or there exists a phase *P* in $\mathcal {T}$ including the 1-edge incident to its sink but not the one incident to its source. This phase *P* forms a matching even when the two flippable red edges are added. Thus, by Lemma 3.2, we can flip *P* also in $\mathcal {O}$. However, *P* contains exactly one of the two 1-edges incident to the minimum and maximum vertices of $\mathcal {O}$. Thus, these 1-edges are not in phase. We conclude that the 1-edges incident to the minimum and maximum vertices are in phase if and only if this also held for both $\mathcal {F}$ and $\mathcal {T}$.

*The* $\exists$ *Quantifier* Now we show how we build a USO $\mathcal {O}:=O_i[b_{1},\ldots ,b_i]$, if $q_{i+1}=\exists$. We again note that $q_{i+1}x_{i+1},\ldots ,q_nx_n:\Phi (b_{1},\ldots ,b_i,x_{i+1},\ldots ,x_n)$ is true if and only if *at least one* of the sentences

$$\begin{aligned} q_{i+2}x_{i+2},\ldots ,q_nx_n:\Phi (b_{1},\ldots ,b_i,B,x_{i+2},\ldots ,x_n) \end{aligned}$$

for $B\in \{0,1\}$, i.e., the two sentences corresponding to $\mathcal {F}:=O_{i+1}[b_{1},\ldots ,b_i,0]$ and $\mathcal {T}:=O_{i+1}[b_{1},\ldots ,b_i,1]$, are true.

**Fig. 7:**
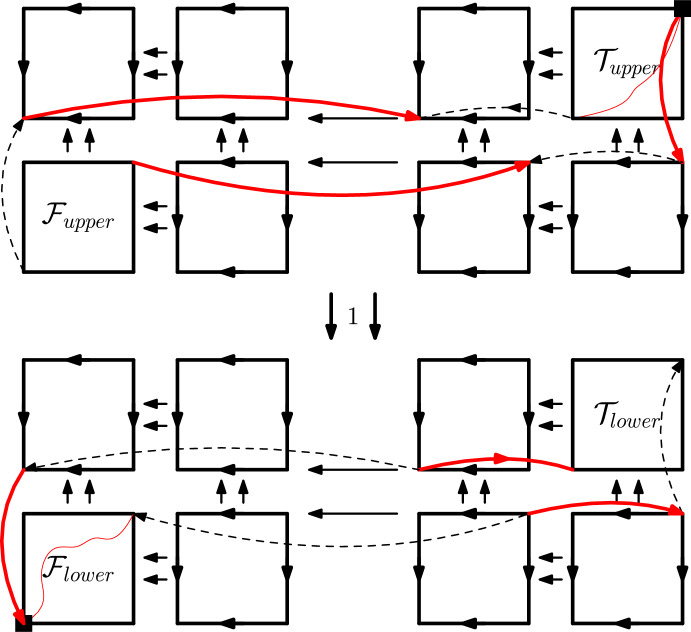
The $\exists$ construction. The 1-edges incident to the square vertices are in phase if and only if the minimum and maximum 1-edges of either $\mathcal {F}$ or $\mathcal {T}$ are in phase

We show how to build $\mathcal {O}$ from $\mathcal {T}$ and $\mathcal {F}$ in Fig. 7. Essentially, $\mathcal {O}$ consists of one copy of $\mathcal {F}$, one copy of $\mathcal {T}$, and six uniform USOs, all connected in a combed way, but then six specially marked red edges are flipped. These edges again connect minimum or maximum vertices of either $\mathcal {F}$ or $\mathcal {T}$ and one of the uniform USOs. Note that by the inductive hypothesis, we can again assume both $\mathcal {F}$ and $\mathcal {T}$ to fulfill the conditions outlined above. We now prove that $\mathcal {O}$ must fulfill the invariants in a very similar way to the proof for the $\forall$-gadget above.

We first prove that $\mathcal {O}$ is a USO: Similarly to the $\forall$ construction, we only need to show that all the red edges are flippable. Again, this follows from the location of the sources and sinks of $\mathcal {F}$ and $\mathcal {T}$. Furthermore, the red edges form a matching, so they can also all be flipped together.

Next, we see that the construction preserves acyclicity: We can again decompose $\mathcal {O}$ into subcubes along combed dimensions. In the remaining subcubes, we see that all red edges are incident to sinks or sources, and can thus not be used in directed cycles. It follows that $\mathcal {O}$ is acyclic.

It is again obvious to see that $\mathcal {O}$ is combed downwards in dimension 1, and the global sink and source of $\mathcal {O}$ are located in the minimum and maximum vertex, respectively.

Finally, we show that the 1-edges incident to the minimum and maximum vertices of $\mathcal {O}$ are in phase if and only if this holds for *at least one of* $\mathcal {F}$ and $\mathcal {T}$.

The “if” direction is again simple, since we can use the in-phaseness sequence through $\mathcal {F}$ and the three lower red and dashed edge pairs, or the in-phaseness sequence through $\mathcal {T}$ and the upper three red and dashed edge pairs.

For the “only if” direction, we see that all 1-edges outside of $\mathcal {F}$ and $\mathcal {T}$ are flippable, except the four that are adjacent to a red edge. We can check all pairs of remaining 1-edges for possibly being in direct phase and verify that only the direct-in-phaseness relations induced by the red and dashed edge pairs and the relations inside of $\mathcal {F}$ and $\mathcal {T}$ are present. Thus, the minimum and maximum 1-edges of $\mathcal {O}$ can only be in phase, if that holds for $\mathcal {F}$ or $\mathcal {T}$.

*Final Arguments* It only remains to prove that we can build a circuit computing the outmap function $O_0[]$ from the QBF instance in polynomial time. Based on the sequence $q_1,\ldots ,q_n$ of quantifiers, we can easily assign the dimensions of $O_0[]$ to the different levels of the recursion (the first three coordinates belong to the base gadgets, and the following coordinates belong to levels in either groups of two or three coordinates, depending on whether $q_i=\exists$ or $q_i=\forall$). We can thus easily locate a given vertex *v* within all levels of the recursive construction. If at some point the vertex is part of a uniform subcube, it does not need to be located on lower levels. Otherwise, the vertex is part of a base gadget $O_n[b_1,\ldots ,b_n]$ on the last level of the recursion. Here, we can evaluate $\Phi (b_1,\ldots ,b_n)$ and find the orientation of *v*. $\square$

## Data Availability

There is no experimental data associated with this paper.
